# A Renal Abscess Caused by Nontyphoid Salmonella in an Immunocompetent Adult

**DOI:** 10.7759/cureus.35749

**Published:** 2023-03-04

**Authors:** Tamar Didbaridze, Giorgi Kochiashvili, Davit Kochiashvili, Irakli Tortladze, Mariam Oniani, Zurabi Zaalishvili, Giorgi Maziashvili

**Affiliations:** 1 Clinical Microbiology, Tbilisi State Medical University the First University Clinic, Tbilisi, GEO; 2 Microbiology, Tbilisi State Medical University, Tbilisi, GEO; 3 Urology, Tbilisi State Medical University the First University Clinic, Tbilisi, GEO; 4 Radiology, Tbilisi State Medical University the First University Clinic, Tbilisi, GEO; 5 Faculty of Medicine, Tbilisi State Medical University, Tbilisi, GEO

**Keywords:** urinary tract infection, urine, atypical infection, salmonella infection, abscess

## Abstract

Renal abscesses are uncommon in adults and are typically caused by gram-negative bacteria such as *Escherichia coli*, *Proteus mirabilis*, or *Staphylococcus aureus*. Nontyphoid *Salmonella* abscesses are infrequent. We discuss the case of a 27-year-old previously healthy female who developed a renal abscess due to *Salmonella enterica*. Abdominal computed tomography (CT) revealed a left renal abscess (size 11.6 cm × 8.2 cm) and 1.9 cm cyst in the right kidney. The urine and abscess aspirate cultures indicated the presence of gram-negative bacilli and lactose-negative *Salmonella*. A presumptive diagnosis of the left renal abscess was made. A urologist was consulted, and the patient was prepared for a left-sided nephrectomy. The patient's condition improved following treatment with a combination of piperacillin/tazobactam and moxifloxacin. Based on our experience, prompt recognition of nontyphoidal Salmonella as a potential cause of a renal abscess is important to prevent complications such as the extension of the abscess and the spread of the pathogen to adjacent structures.

## Introduction

Around 550 million people are infected with Salmonella every year [1], making it a significant pathogen. Nontyphoid Salmonella (NTS) serotypes infect a wide range of hosts, giving symptoms that vary from mild gastroenteritis to severe systemic infection. Typhoid fever is caused by typhoid Salmonella (TS) serotypes typhi, paratyphi A and B, which have adapted to humans. Nontyphoidal salmonellae are pathogens that can be found in food and water [2,3] and cause gastroenteritis, bacteremia, and focal infection. NTS has a growing influence on human health, accounting for approximately 93.8 million infections and 155,000 fatalities every year [1,3]. Reports of renal abscesses caused by NTS or *Salmonella typhi* are uncommon and typically occur in patients with urologic problems, immunosuppression, or diabetes [4]. In general, antibiotics are used to treat renal abscesses, albeit surgical intervention may be required in some circumstances. In both standard hospital practice and scholarly research, urinary tract infections (UTIs) brought on by Salmonella are rare. However, both immunocompetent and immunocompromised patients may experience significant morbidity as a result. In laboratory practice, Salmonella isolation from non-enteric samples is less frequent [5]. Microbiological culture is an essential procedure for diagnosing sepsis. However, the use of antibiotics prior to sampling frequently decreases the rate of bacterial detection in culture investigations. Very few studies have reported the occurrence of NTS renal abscesses. We report a case of a female who was found to have a left renal abscess caused by *Salmonella enterica.*

## Case presentation

On September 29, 2022, a 27-year-old female with no significant past medical history was admitted to the Tbilisi State Medical University (TSMU) First University Clinic with left flank pain radiating towards the left inguinal region, fever, nausea, vomiting, and generalized weakness. She stated that she had experienced the same symptoms intermittently over the last month, but that the pain had become aggravated and constant in the last five days. On admission, she was febrile with a temperature of 39.0°C (102.0°F) and stable vital signs. Aside from left costovertebral angle tenderness and suprapubic tenderness, no other abnormal examination findings were noted. The patient's medical history did not reveal any chronic conditions, and she does not take any medications regularly. The family history was also unremarkable.

As seen in Table 1, laboratory results at the time of admission showed lymphopenia (1.22 × 10^9^), neutrophilia (14.81 × 10^9^), and leukocytosis (18.24 × 10^9^) as well as elevated levels of C-reactive protein (272), international normalized ratio (1.43), and creatinine (106.4 mol/L). Urinalysis was positive for leukocyturia, hematuria, and proteinuria +3.

**Table 1:** Abnormal laboratory values. CRP: C-reactive protein; INR: international normalized ratio.

	Patient’s lab values	Normal lab values
Lymphocytes	6.70%	20-40%
Neutrophils	81%	40-60%
Leukocytes	18.24 × 10^9^/L	4.5 × 10^9^/L-11 × 10^9^/L
CRP	272 mg/dL	<10 mg/dL
INR	1.43	<1.1
Creatinine	106.4 mg/dL	0.6-1.1 mg/dL

**Figure 1:**
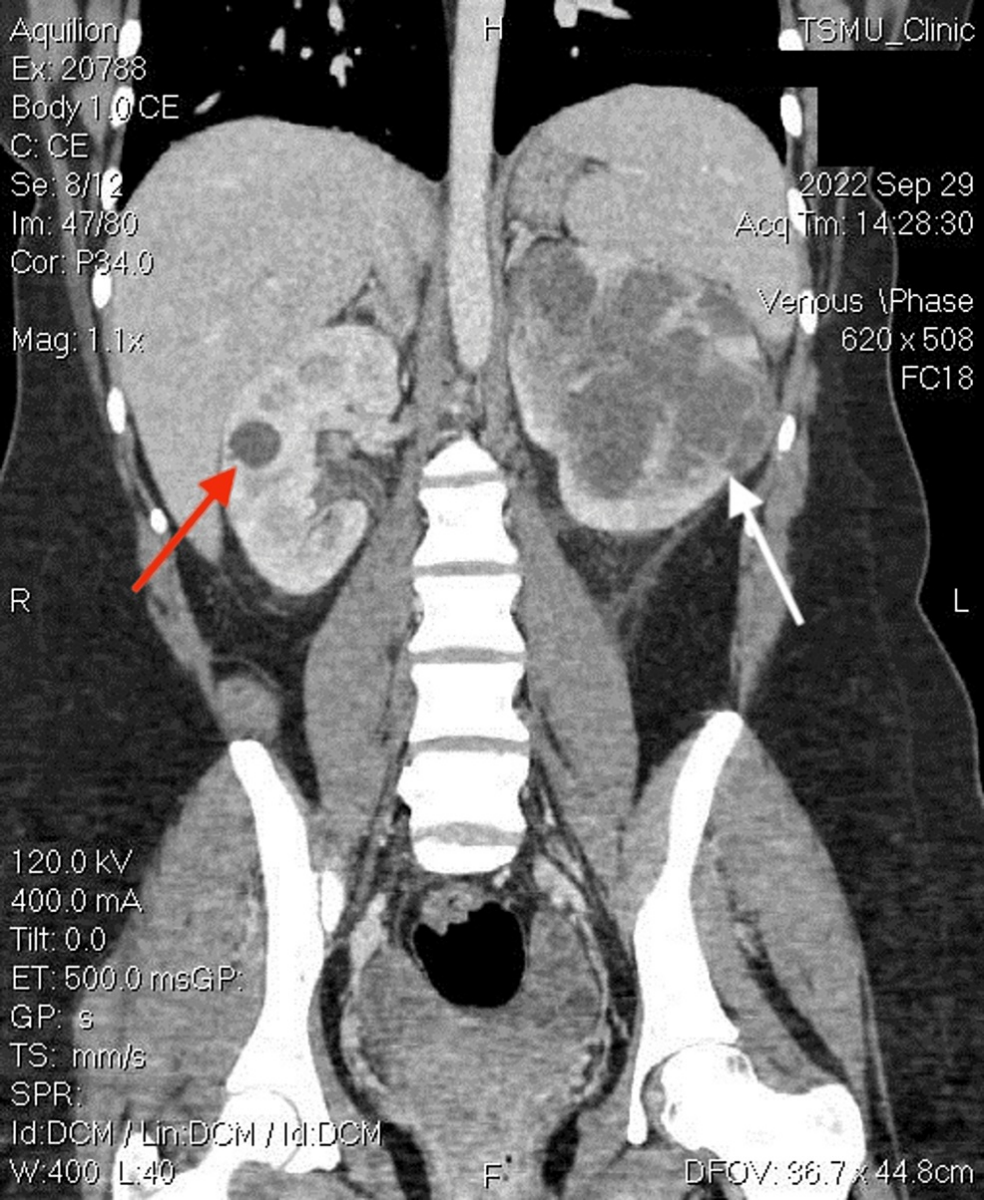
Coronal view of CT scan showing left-sided loculated renal abscess (white arrow) and right-sided renal cyst (red arrow). White arrow: left-sided loculated renal abscess. Red arrow: right-sided renal cyst. CT: computed tomography.

**Figure 2:**
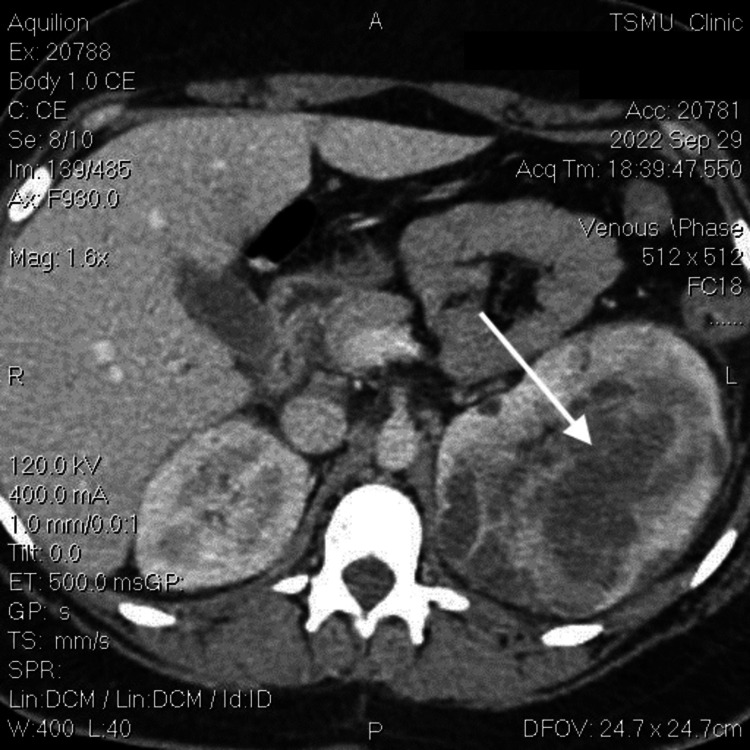
Axial view of CT scan showing left-sided loculated renal abscess (white arrow). CT: computed tomography.

As depicted in Figures 1, 2, abdominal computed tomography (CT) revealed a left renal abscess (white arrow) and 1.9 cm cyst in the right kidney (red arrow). On the left side, the upper pole of the kidney shows an inhomogeneous echotexture. Within the parenchyma, there is a multi-chambered, septated mass measuring 11.6 × 8.2 cm with an extensive fluid density that extends subcapsularly. In addition, there is evidence of a zone of perifocal infiltration within the kidney parenchyma. These observations strongly suggest an abscess. On the right side of the renal parenchyma, cystic foci measuring up to 1.9 cm are observed. The renal pelvis is not dilated, and the adjacent paranephric fat is mildly infiltrated. There are no signs of obstructive uropathy.

A presumptive diagnosis of the left renal abscess was made. After consulting with a urologist, the patient underwent a left-sided nephrectomy, which confirmed the presence of an abscess. Urine and abscess aspirate cultures were ordered, and both grew non-lactose fermenting gram-negative bacilli that were identified as *Salmonella* spp. using the manual identification system of the analytical profile index (API) 20E. Serotyping was performed by the reference laboratory of the National Center for Disease Control and Public Health’s (NCDC), which confirmed *Salmonella enterica*. Kirby-Bauer disk-diffusion method and the European Committee on Antimicrobial Susceptibility Testing (EUCAST) standard version 12 were used to assess antibiotic susceptibility against ampicillin-sulbactam, ceftriaxone, ciprofloxacin, levofloxacin, moxifloxacin, amikacin, meropenem, nitrofurantoin, trimethoprim/sulfamethoxazole. As shown in Table 2, the discovered pathogen exhibited no pattern of resistance to any of the tested antibiotics.

**Table 2:** Antibiotic susceptibility testing using the disk diffusion method (EUCAST guidelines). EUCAST: European Committee on Antimicrobial Susceptibility Testing.

Antibiotics (generic)	Interpretation
Ampicillin-sulbactam	Sensitive
Ceftriaxone	Sensitive
Ciprofloxacin	Sensitive
Levofloxacin	Sensitive
Moxifloxacin	Sensitive
Amikacin	Sensitive
Pefloxacin	Sensitive
Nitrofurantoin	Sensitive

Patient was transferred to the postoperative care unit, where the treatment plan included intravenous piperacillin + tazobactam 4.5 g, intravenous moxifloxacin 400 mg, subcutaneous enoxaparin 40 mg/0.4 mL, oral omeprazole 20 mg. On the 12th day of hospitalization, the patient's symptoms had improved, and she was discharged with a prescription for moxifloxacin (400 mg, once daily) for one week and subcutaneous enoxaparin injection (40 mg/0.4 mL) for ten days.

## Discussion

Renal abscess is a severe complication caused by a bacterial infection in the kidney. One type of bacteria that can rarely cause a renal abscess is nontyphoidal Salmonella [4]. It is most commonly seen in individuals who have underlying medical conditions that increase their risk of infection, such as diabetes [6]. Our patient did not have a recorded history of any of the chronic diseases that could have predisposed her to the infection. Since she was from a suburban area of Georgia, we believe she may have consumed contaminated water or food, which could be the source of the infection. Salmonella may result in bacteremia, gastroenteritis, and subsequent localized infections. In contrast, invasive nontyphoidal salmonellosis seldom affects the kidneys, and renal abscesses produced by Salmonella serovars such as* S. virchow* [7], *S. enteritidis*, *S. typhimurium* [8,9], and *S. oranienburg* [10] have been reported infrequently.

Symptoms of a renal abscess caused by nontyphoidal Salmonella can include fever, chills, and abdominal pain [11]. In addition, individuals may also experience nausea, vomiting, and an overall feeling of being unwell. These symptoms were present in our patient, although the main reason she came to the hospital was left flank pain which has been aggravating over the past five days. 

Diagnosis of a renal abscess caused by nontyphoidal Salmonella is usually made with the help of imaging tests, such as a CT scan or an ultrasound [12]. Furthermore, a sample of the infected tissue can be extracted and sent to a laboratory to undergo culture and sensitivity testing. This helps to identify the type of bacteria that is causing the infection and determine the most suitable antibiotics to effectively treat it. In our case, CT revealed a left renal abscess, and antibiotic susceptibility showed no resistance pattern. 

Treatment of small renal abscesses typically involves antibiotic therapy [13]. For larger renal abscesses, antibiotics are used to prevent the spread of infection, while surgery is required to drain the abscess and remove any dead tissue [14]. As described in our case, the patient was given a combination of IV antibiotics post-nephrectomy and was discharged on day 12 with a one-week prescription for moxifloxacin.

## Conclusions

A previously healthy young female presented with a left renal abscess caused by *Salmonella enterica*, which was successfully treated with nephrectomy and appropriate antibiotic treatment. The case demonstrates the importance of prompt identification of uncommon pathogens and susceptibility testing to guide effective antimicrobial therapy. It also emphasizes the significance of careful antibiotic use to reduce the risk of antibiotic-resistant pathogens.
